# Supplementary figures and images for: DBF4, not DRF1, is the crucial regulator of CDC7 kinase at replication forks
Source: J Cell Biol. 2024 Jun 12;223(8):e202402144. doi: 10.1083/jcb.202402144 (PMC11169917; doi:10.1083/jcb.202402144)

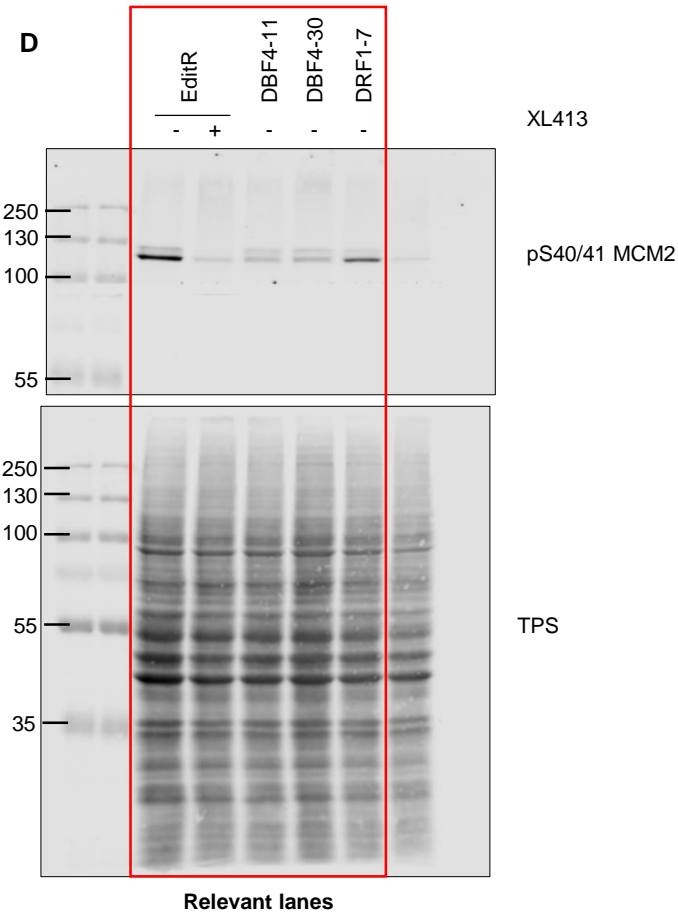

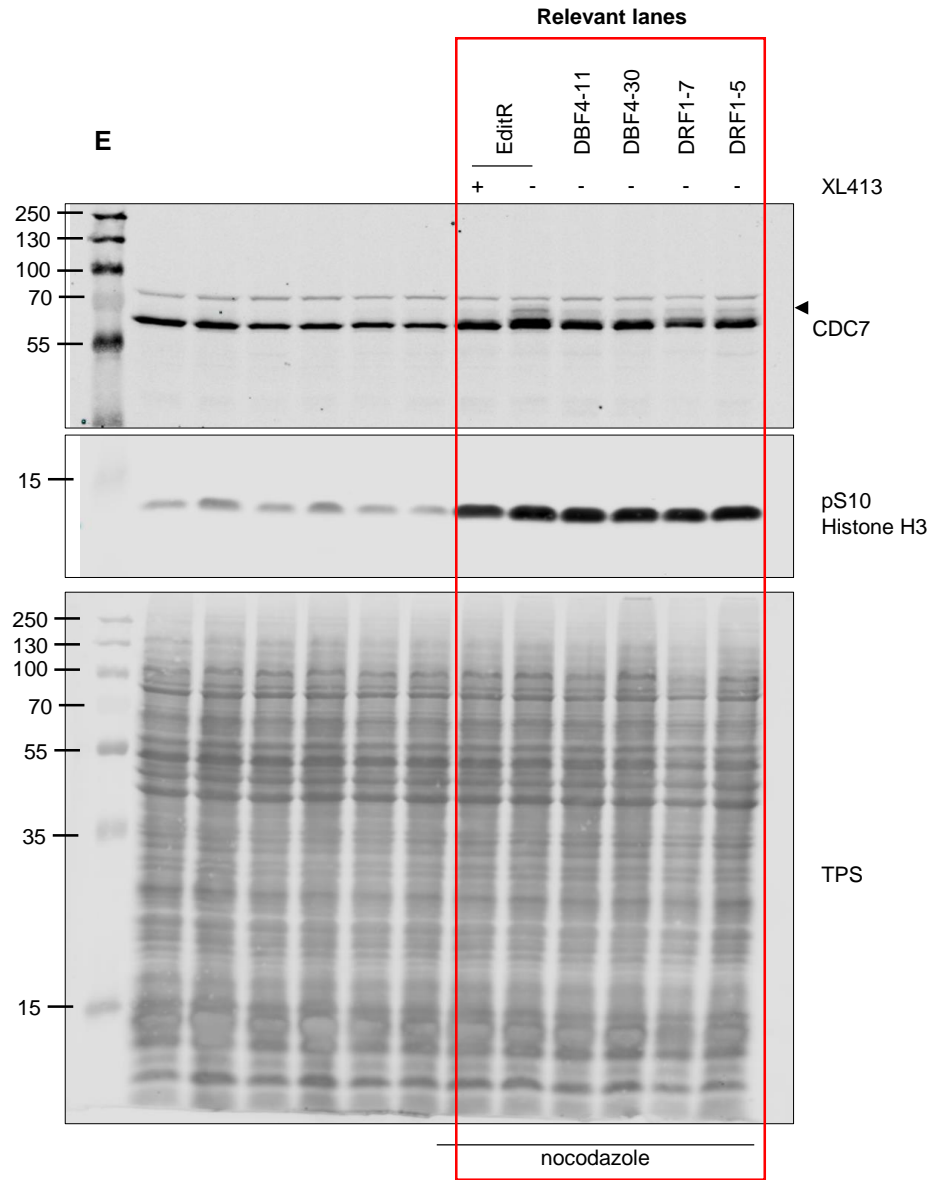

Supplement: SourceData F1 — is the source file for Fig. 1. [file jcb_202402144_sourcedataf1.pdf]

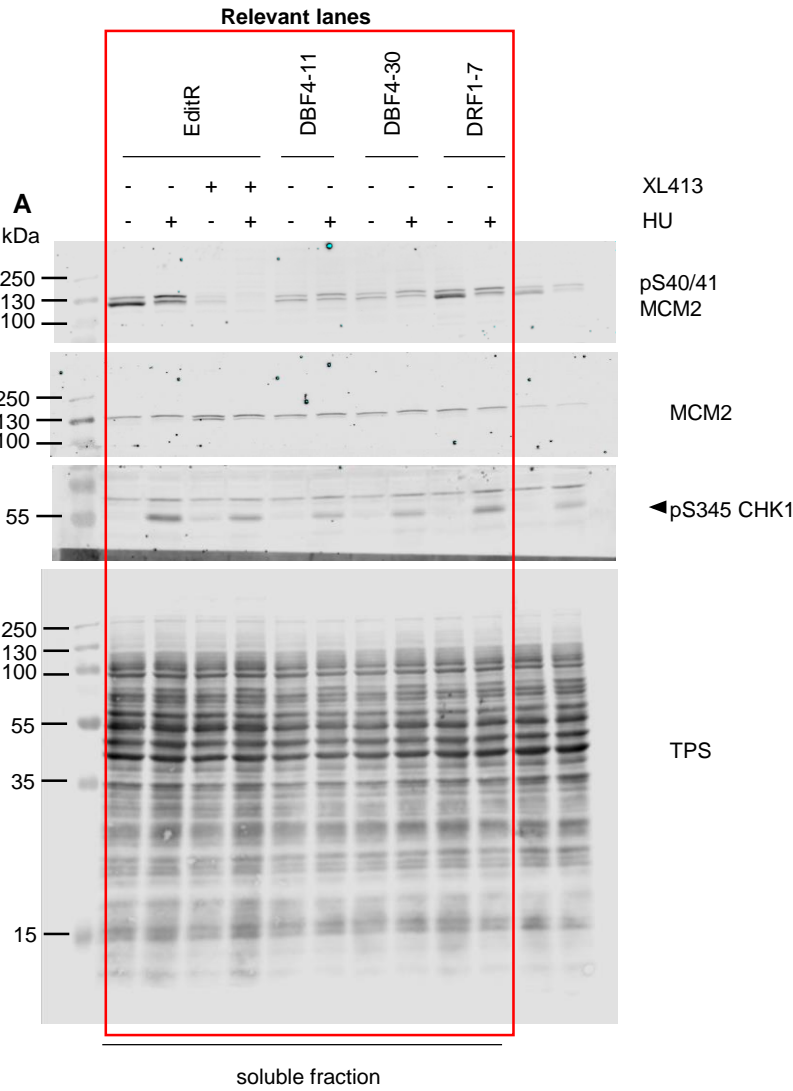

SourceDataFigure 4

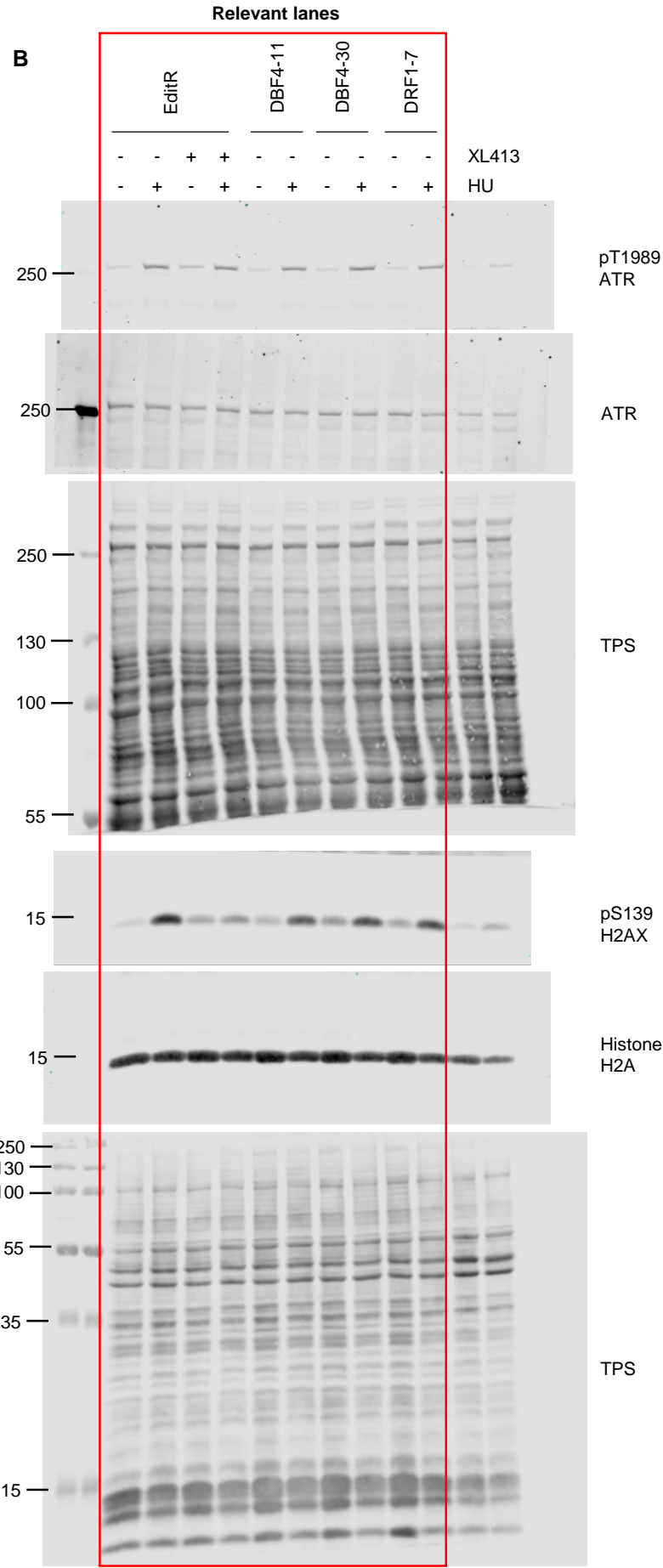

Supplement: SourceData F4 — is the source file for Fig. 4. [file jcb_202402144_sourcedataf4.pdf]

A

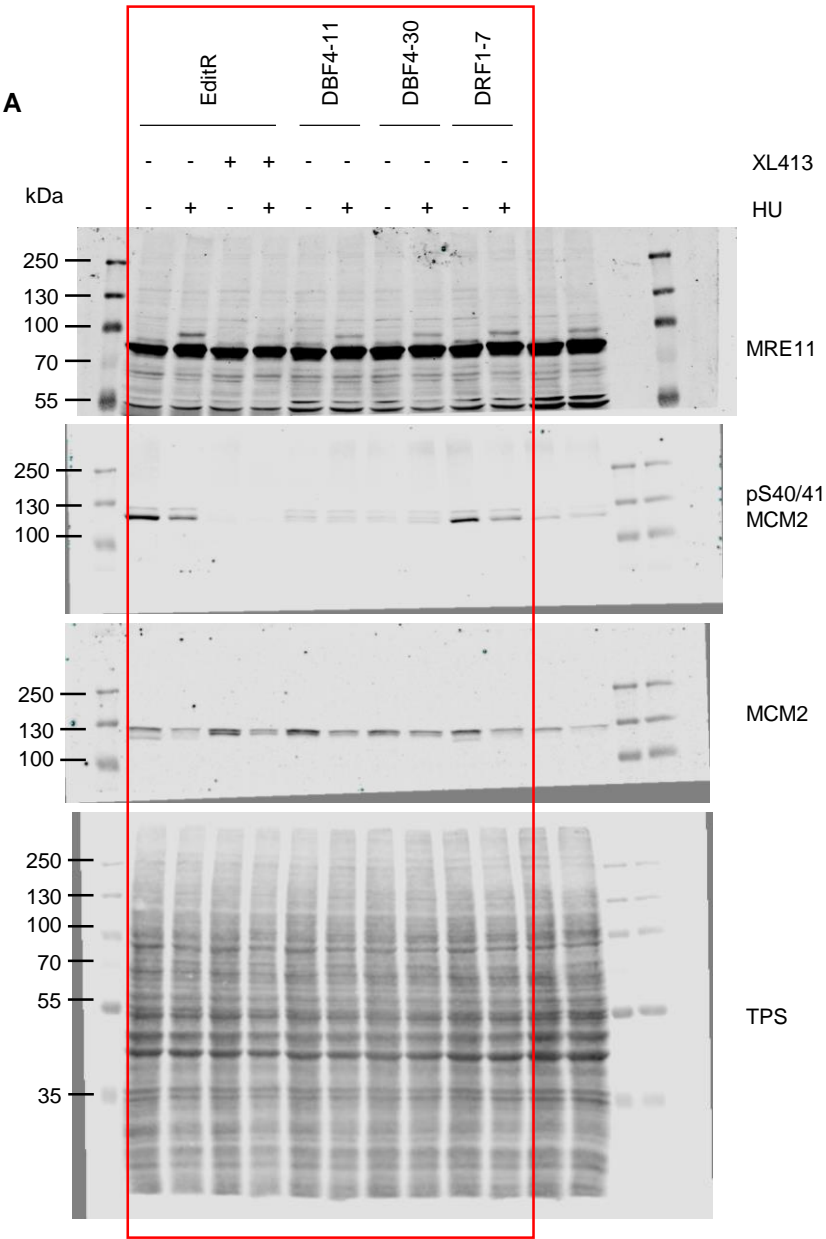

Relevant lanes

C

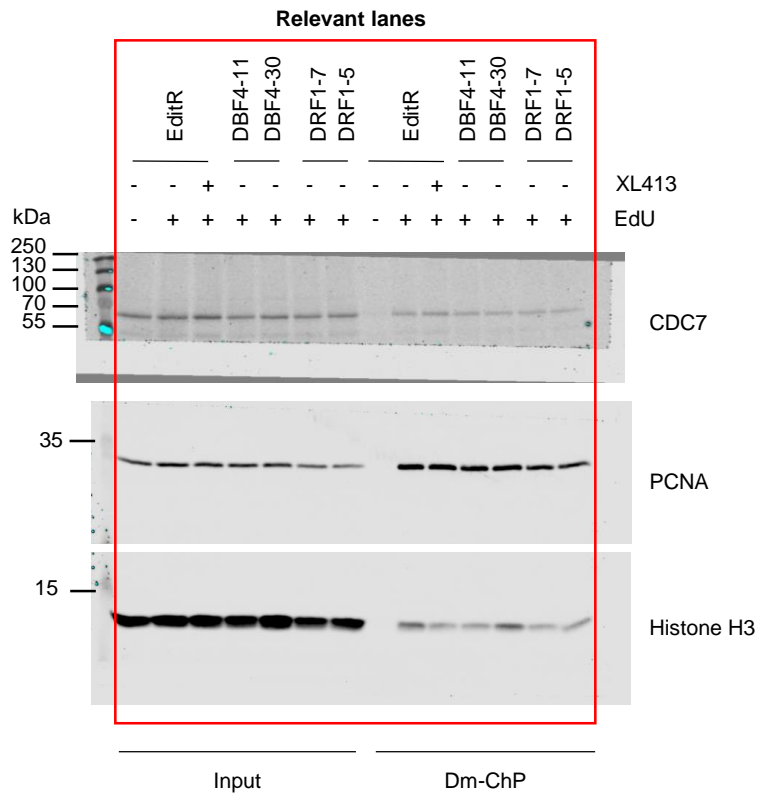

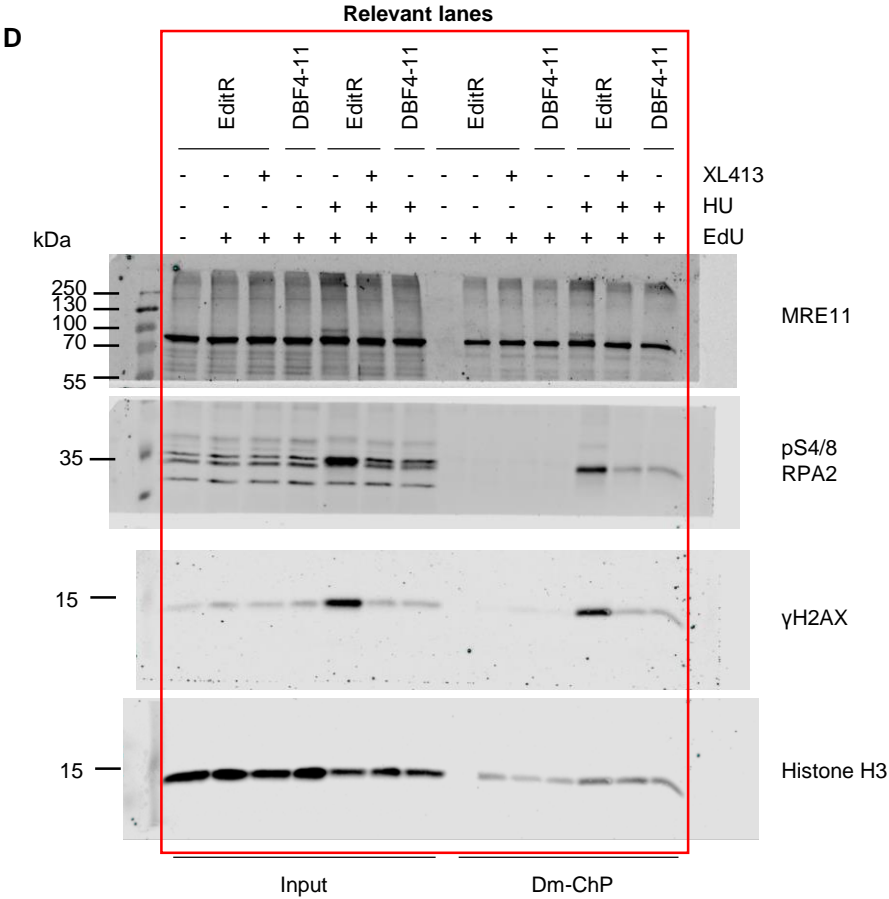

E

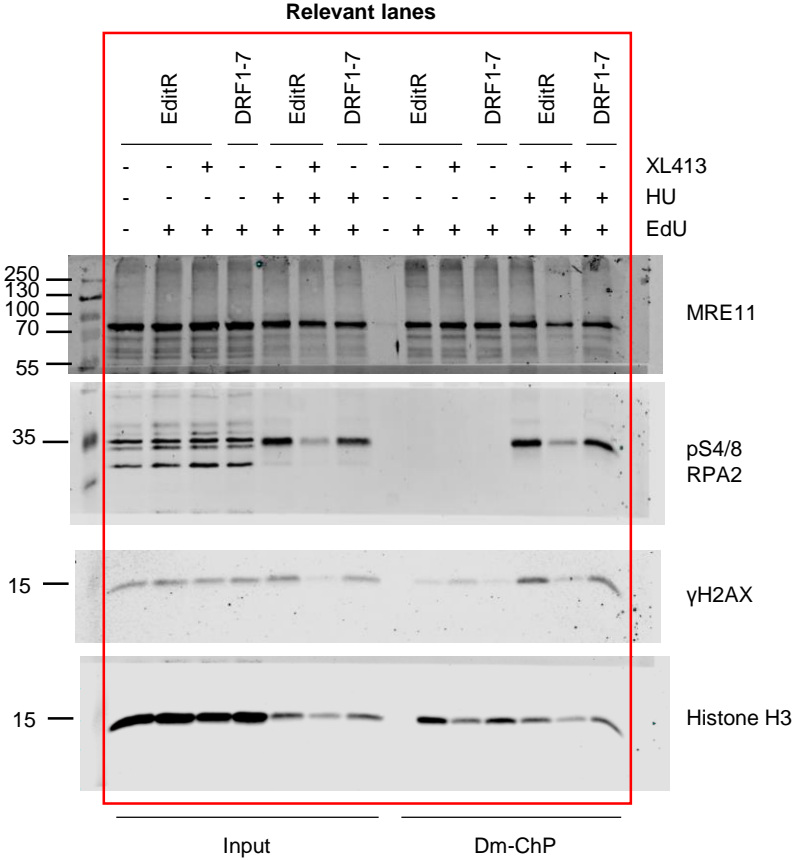

Supplement: SourceData F5 — is the source file for Fig. 5. [file jcb_202402144_sourcedataf5.pdf]

B

Gel1

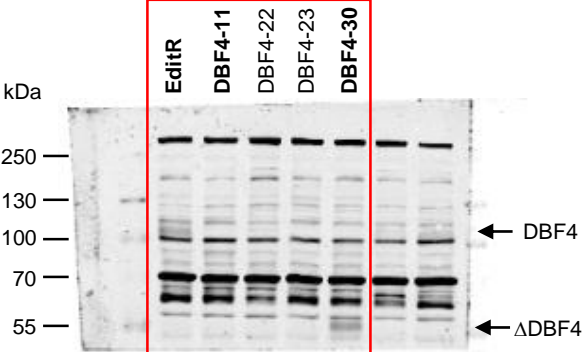

Gel2

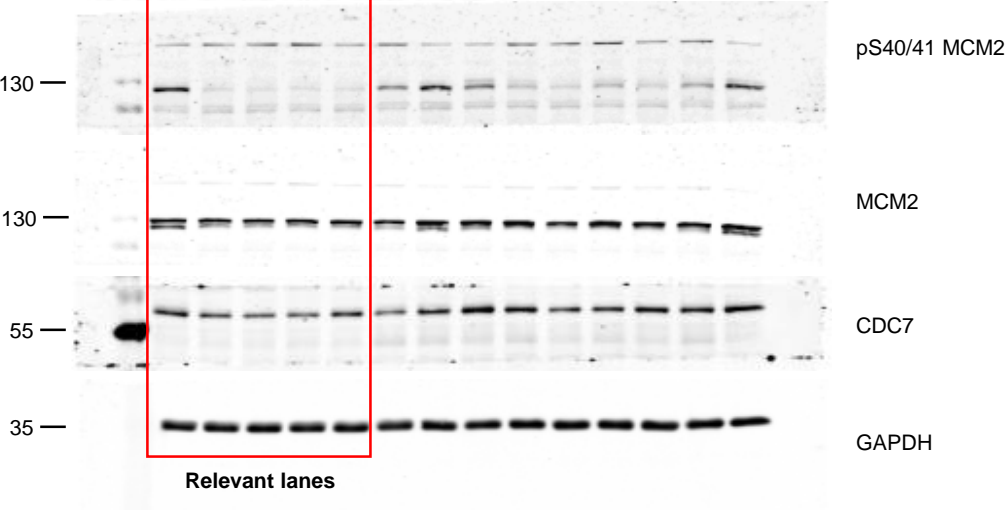

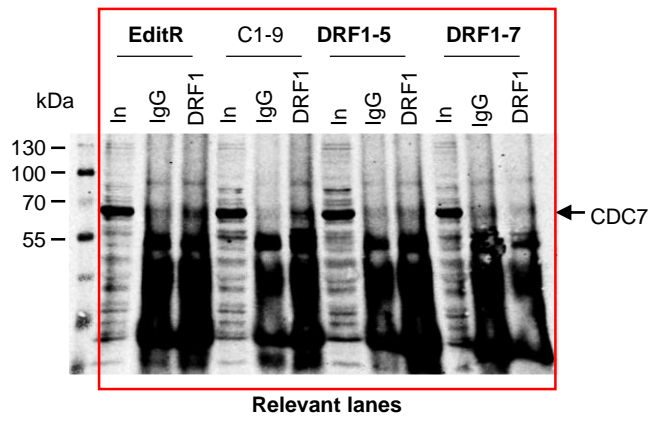

G

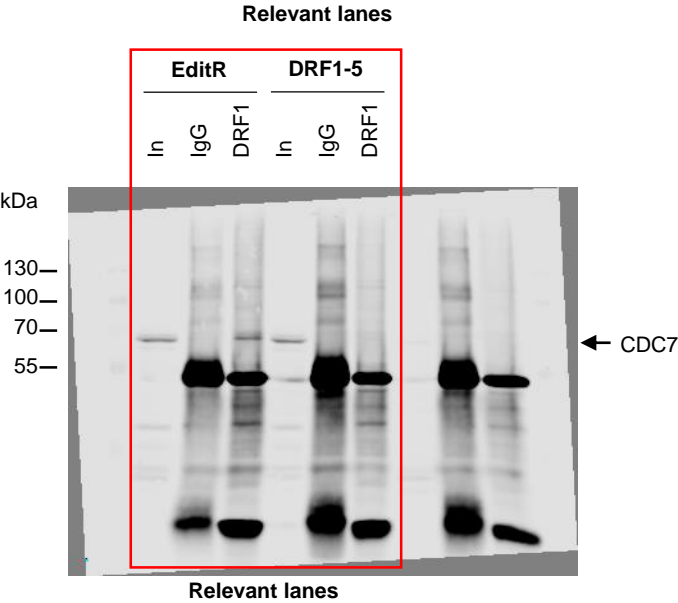

Supplement: SourceData FS1 — is the source file for Fig. S1. [file jcb_202402144_sourcedatafs1.pdf]
